# Discontinuities between maternity and child and family health services: health professional’s perceptions

**DOI:** 10.1186/1472-6963-14-4

**Published:** 2014-01-06

**Authors:** Kim Psaila, Virginia Schmied, Cathrine Fowler, Sue Kruske

**Affiliations:** 1 School of Nursing and Midwifery, University of Western Sydney, Parramatta Campus, Penrith South DC 1797, Penrith, NSW 2751, Australia; 2 School of Nursing and Midwifery and the Family and Community Health Research Group, University of Western Sydney, Parramatta Campus, Penrith South DC 1797, Penrith, NSW 2751, Australia; 3 Child & Family Health Centre for Midwifery, Child & Family Health Faculty of Nursing, Midwifery & Health University of Technology Sydney, Sydney, Australia; 4 Queensland Centre for Mothers & Babies, The University of Queensland, Brisbane, Qld 4072, Australia

## Abstract

**Background:**

Continuity in the context of healthcare refers to the perception of the client that care has been connected and coherent over time. For over a decade professionals providing maternity and child and family health (CFH) services in Australia and internationally have emphasised the importance of continuity of care for women, families and children. However, continuity across maternity and CFH services remains elusive. Continuity is defined and implemented in different ways, resulting in fragmentation of care particularly at points of transition from one service or professional to another.

This paper examines the concept of continuity across the maternity and CFH service continuum from the perspectives of midwifery, CFH nursing, general practitioner (GP) and practice nurse (PN) professional leaders.

**Methods:**

Data were collected as part of a three phase mixed methods study investigating the feasibility of implementing a national approach to CFH services in Australia (CHoRUS study). Representatives from the four participating professional groups were consulted via discussion groups, focus groups and e-conversations, which were recorded and transcribed. In total, 132 professionals participated, including 45 midwives, 60 CFH nurses, 15 general practitioners and 12 practice nurses. Transcripts were analysed using a thematic approach.

**Results:**

‘Continuity’ was used and applied differently within and across groups. Aspects of care most valued by professionals included continuity preferably characterised by the development of a relationship with the family (relational continuity) and good communication (informational continuity). When considering managerial continuity we found professionals’ were most concerned with co-ordination of care within their own service, rather than focusing on the co-ordination between services.

**Conclusion:**

These findings add new perspectives to understanding continuity within the maternity and CFH services continuum of care. All health professionals consulted were committed to a smooth journey for families along the continuum. Commitment to collaboration is required if service gaps are to be addressed particularly at the point of transition of care between services which was found to be particularly problematic.

## Introduction

Evidence is growing on the impact of parenting [1-4] and environmental influences on the long-term development, health and wellbeing of children [5,6]. Governments are committed to the care and protection of children, both in Australia [7,8] and internationally [9]. In Australia, publicly funded, universal health services are available to all pregnant women, children and their families and include pregnancy and birth care, parenting support, developmental monitoring of children, preventative health (e.g. immunisation) and health promotion (e.g. breastfeeding) [10]. However, health professionals, policy-makers and families increasingly express concerns about inconsistent, fragmented health services which often do not meet the needs of the population [11].

Integrated or collaborative service models have been proposed to reduce fragmentation [12]. These models aim to improve continuity within and across services and are viewed as more effective and economical in delivering services to families [8,13]. Despite the potential advantages of integrated services [9,14], challenges to implementation and the role of individual health professionals should not be underestimated [9,15,16].

The Child Health: Researching Universal Service (CHoRUS) study, is a three phased mixed methods study investigating the feasibility of implementing a national approach to the provision of universal services to children and families. This paper examines the concept of continuity across the maternity and the child and family health (CFH) service continuum from the perspectives of professional leaders in midwifery, child and family health (CFH) nursing, general practice and practice nursing.

## Background

Australia provides universal CFH services within a framework of primary health care [10,11,17]. The universal health service commences antenatally through contact with publicly funded maternity services provided predominantly by midwives. After discharge from hospital, in most Australian States and Territories, postnatal care comprises a home visit from a midwife and/or the CFH nurse within two weeks [18]. Additional contact points occur over the next five years, the timing of which varies considerably between jurisdictions, and is undertaken by CFH nursing services and to a lesser extent, general practitioners (GP) [11,19]. Nurses employed within CFH nursing services have a different nomenclature in most states and territories for example, CFH nurses, maternal and child health nurses, child health nurses. In this paper we have used the title of CFH nurse as this is the most common title used nationally.

While general practice also provides substantial child healthcare [20], limited literature exists on the role GPs play in providing well child health care [21]. Despite the emphasis placed on preventative health measures [22], elements of the universal CFH service schedule are generally only provided opportunistically by GPs during visits for immunisation and illness [21].

Targeted or secondary services are provided for children and their families with additional needs. For example, sustained nurse home visiting programs are aimed at families where infants are less likely to achieve optimal outcomes [23,24]. In addition, specialist or tertiary services are available for children with acute or high-level needs, who would otherwise be at high risk for poor health and development outcomes. Until recently no consistent framework for the provision of universal CFH services existed nationally [11]. While the current framework recommends key ages and developmental stages when universal health services should be delivered to families, no specific time points are stipulated [10].

Empirical research and reviews have identified several problems with the provision of universal CFH services in Australia [6,21,25]. These include ineffective communication pathways and restricted information transfer; limited collaboration between professionals and across services; unclear role boundaries; and inadequate professional education and training to support pregnant women, children and families with complex needs [6]. Concerns about lack of continuity and fragmentation in service provision have prompted governments at all levels to promote service integration to better meet the needs of developing children and provide support to families [12,26-28]. Homer et al. [29] found a range of models in place within one Australian state to facilitate continuity at the point of transition from maternity to community-based CFH services. Some were structured but many had developed in an evolutionary or ad hoc way depending on local context, expertise, interests and policies [29]. Discontinuity is most likely to occur at points of transition of care, when the care of a woman or family is transferred between services or between health providers.

### Continuity

Reid et al. [30] describe continuity as the degree to which a series of discrete events are experienced as coherent, connected and consistent with the patient’s needs over time. They maintain that two core elements are essential to continuity: that care is experienced as smooth and coordinated by the client, and is provided over time. However, the presence of these two elements alone does not constitute continuity [30].

Reid et al. [30] also identified three intertwining dimensions within the concept of continuity of care: informational continuity; relational continuity; and management continuity. Informational continuity refers to how information relating to a patient’s previous history is used to inform current care planning. Knowledge of the patient’s values, preferences and social context is as equally important as knowledge of physical condition. Relational continuity focuses on the clinician – client relationship developed over time which provides a framework for more consistent care. Management continuity refers to co-ordination of patient care, and how it is organised and provided via timely and consistent service, appropriate to the needs of the individual. This term usually refers to interactions between services or providers.

The concept of continuity is important to maternity and CFH service professionals but has been defined and applied in varying ways. The term ‘continuity of carer’ has been used extensively to refer to care provided to a woman by one or a small team of known midwives during pregnancy, birth and the postnatal period [31-33]. General practice employs the term ‘therapeutic relationship’ or ‘the medical home’ to refer to the relationship between the client and GP as the consistent carer [34]. The Royal Australian College of General Practitioners (RACGP) 2010 standards adopt Reid et al.’s [30] dimensions of continuity, stating that these are achievable for general practice in Australia, without discussing how this is to be achieved. While in some instances ‘therapeutic relationship’ or ‘the medical home’ may refer to the client consistently being seen by a single practitioner, continuity may also be interpreted to mean the client consistently attends the same service and may see any one of several practitioners. While either option would support informational continuity, the other dimensions of continuity would differ. The way in which GPs collaborate with other professionals, services and agencies remains unclear [35].

CFH nurses are directed via policy and professional competency statements to collaborate with other health professions to provide continuity of service [8,36,37]. Yet, very little literature addresses the nature of continuity in CFH nursing practice; what does exist reports gaps in continuity of care between CFH nurses and other maternity and CFH professionals [29,38].

A service continuum implies the existence of continuity across the individual services spanning the continuum. The maternity and CFH service continuum is complex, spanning maternity services (private and public), CFH nursing services and general practice. This leads to potential fragmentation and service discontinuity, particularly at the transition of care from maternity services to CFH services and general practice [10,29]. While research has identified facilitators and barriers to continuity between services [30,39], very little has explored continuity of care along the maternity and CFH service continuum.

This paper examines the concept of continuity across the maternity and CFH service continuum from the perspectives of midwifery, CFH nursing, general practitioner (GP) and practice nurse (PN) professional leaders. Data reported in this paper was collected in phase one of the CHoRUS study. The results of phases two and three will be reported in subsequent papers.

## Methods

### Research design

The CHoRUS study used a three phase, mixed methods sequential design [40] to investigate the feasibility of implementing a national approach to the provision of universal services to children and families. Burke Johnson, et al. [41] describe mixed method research as an approach to knowledge that attempts to consider multiple viewpoints, perspectives, positions and standpoints [41].

A pragmatic philosophical approach was used for the study as it challenges the notion of a single absolute truth being attainable [42-44]; it proposes that truth is subjective and variable rather than absolute. The statement of the problem therefore determines how one goes about solving it and the methods used should be individually tailored to the problem [42-44]. The CHoRUS study used quantitative and qualitative methods to investigate the complexities of the maternity and CFH nursing continuum, facilitating rigorous analysis by balancing the strengths and weaknesses of each approach [45].

The first phase explored the current status of maternity and CFH service delivery, including facilitators and barriers to service provision across the maternity and CFH continuum of care. A qualitative approach to data collection and analysis was therefore deemed appropriate in order to capture the experiences of midwives and CFH nurses providing universal services to women and families in Australia.

The Human Research Ethics Committee at the University of Western Sydney (UWS), a registered NHMRC HREC approved the study.

### Participants and recruitment

We invited representatives from each of the professional groups involved in the delivery of universal and/or primary care services for pregnant women, well children and their families (midwives, CFH nurses, GPs and PNs) to participate. We considered professional leaders to be those professionals who were active in their professional group or were nominated by their respective professional organisation to participate in the study.

We recruited participants through the relevant professional organisations: Australian Association of Maternal, Child and Family Health Nurses (formerly AAMCFHN now MCaFHNA), Australian College of Midwives (ACM), Royal Australian College of General Practitioners (RACGP), Australian General Practice Network (AGPN) and Australian Practice Nurse Association (APNA). Initially we requested the organisations to nominate professional leaders as representatives. Potential participants identified by each association were emailed information about the study, the questions to be asked and a consent form; those who agreed returned consent forms prior to participating.

In total 60 CFH nurses were consulted, 42 via discussion groups held at a national conference and an additional 8 CFH nurses via a teleconference and 10 in a face to face group. Forty-five midwives were also consulted, 40 via two face-to-face focus groups and five via teleconference. Given the difficulties organising face-to-face focus groups with GPs, we collected data via email (referred to as e-conversation) with nine members of the RAGCP. GPs provided, via email, feedback to the open-ended questions used in the focus groups with other professionals. In addition, we conducted a teleconference with six representatives from the AGPN. We held two teleconferences with 12 practice nurses working within general practice with assistance from the APNA.

### Data collection

The focus groups and teleconferences ranged in duration from 60 to 90 minutes. Two or three researchers attended each focus group or teleconference. One researcher led as group facilitator, while the other researcher/s took notes and observed group interaction. Questions were tailored for each professional group. All consultations were digitally audio recorded with participants’consent.

### Data analysis

Data from the consultations were transcribed verbatim and imported into QSR NVivo 9.1 for analysis. We developed an *apriori* coding template based on the relevant literature. The template provided a starting point and was not intended to be an exclusive list of codes; additional codes were applied as required [46]. This template included the three intertwining dimensions within continuity of care: informational continuity; relational continuity; and management continuity [30].

We analysed the data thematically, informed by Reid et al’s [30] concept of continuity and Braun and Clarke’s [47] 6-step process of thematic analysis. This process facilitated the recording of a systematic, clear and reproducible audit trail. The first author read all transcripts repeatedly to ensure complete familiarisation and then undertook systematic initial coding, with ideas and patterns allocated to as many codes as they fit. Data extracts were coded inclusively to preserve the context in which each comment was made. The researcher also recorded memos containing additional observations and reflections. The second author read all transcripts to confirm the identification of themes and concepts.

Initial coding identified 49 codes, which were then developed into 21 concept groups. For example the concept of ‘service delivery’ embraced the codes: ‘duplication of service’, ‘transition of care’, ‘continuity of care’, ‘visible’ and ‘accessible’. We then examined the data to develop a coding map looking for patterns or linkages in the data highlighting potential themes related to the research question.

## Results

The analysis identified continuity as a major theme in the data. It also identified and confirmed three subthemes contributing to continuity: ‘developing a relationship with the family’, ‘communication pathway’ and ‘co-ordination of care’. These can be related to the constituent dimensions in Reid et al’s framework [30].

### Continuity

The meaning of continuity varied across and within professional groups. While most participants perceived continuity to refer to a woman or family consulting the same health professional each visit (continuity of carer), other participants provided examples of a service definition of continuity, when families are seen by a variety of professionals working within the same service (continuity of service).

Continuity was a term used by each of the professional groups to describe the ‘ideal’ for families and was proposed by all professional groups as their rationale for attempting to develop a relationship with women and families. Respondents believed that developing a relationship with families would enable them, through successive consultations, to facilitate ongoing support for maternal health, and child health and development.

The midwives placed a great deal of significance on continuity, which they perceived as being the development of a relationship with the woman built over time, through continuity of the midwife as the primary carer (continuity of carer). This emphasis on continuity may explain why, in some services, midwives actively engaged with CFH nurses. The midwives reported advantages for families of the CFH nurse meeting and introducing their service during the antenatal period:

…introduce them to some of the [CFH] nurses while they’re in that environment so when they have got a baby they know exactly where they need to go and that sort of thing. Then when we’re doing their postnatal visits we talk to them about what will happen when we refer them on to [CFH services]. So we’re getting them ready for that transition during their pregnancy as well. [Midwife ACM]

In contrast, CFH nursing promotes a continuity of service model. Participants indicated that in most instances a CFH nurse is allocated to a family for the initial visit. But, if required, subsequent visits were typically, although not always, undertaken by different CFH nurses. This model emphasised that while different nurses may work with families, the philosophy of care was consistent. However, many CFH nurse participants still viewed continuity as the midwives did, as an ongoing relationship developed over time through continuity of carer.

I think that continuity of relationship is so important for the families and for us as well, because we actually develop that knowledge base which we then build on with them. [CFH nurse MCAFHNA]

While individual CFH nurses might focus their energies on developing trusting relationships with families, the organisational view was that the relationship should be developed with the service. Many CFH nurses identified this philosophical change in policy as being imposed upon them and at odds with the relationship based model of care they were also encouraged to work within. Several CFH nurses expressed finding the mismatch difficult:

One of the big challenges we have … is that the person or nurse who does all the universal contact home visit often never sees the parent again. It’s a one off. That’s it. So the whole premise of establishing a relationship has been argued from the service to be establishing a relationship with the service. Not establishing a relationship with the individual. [CFH nurse MCAFHNA]

General practitioners typically characterised their service as providing ‘relational continuity’ with a consistent care provider (continuity of carer). However, participants reported that, as practices become busier, families often see multiple practice members rather than “their own doctor”; GPs increasingly find themselves providing “one off care” to families.

…now we see a lot of GP clinics that are not unlike road side service stations where people come in as a convenience - totally oblivious to the notion of continuing care. [GP RACGP]

Each professional group viewed continuity in terms of continuity *within* their own service rather than continuity *between* services. Therefore they placed considerable importance on the impact of informational, relational and managerial continuity on their relationship with individual families, as shown in the following sub-themes.

### Developing a relationship with the family: relational continuity

All professional groups saw the *development of a relationship* with the family as crucial for continuity. Although often contradicting the organisational philosophy of service continuity, participants perceived that the development of an individual carer relationship with the family would increase the family’s likelihood of continuing to access the professional’s service and participating in their own care planning. This in turn ensured the professional an opportunity to continue surveillance of the children’s health and development and parental well-being.

Midwives believed it was important to develop a “good” relationship with women and their families in order to provide quality care and to present the health service in a positive light, encouraging families to maintain contact.

If they have a positive experience with us then we can help sell the next service. [Midwife ACM]

Midwives commented on the strength of the woman/midwife relationship developed within a continuity of carer midwifery model. Despite the advantages for women and midwives, this relationship may hinder the transition of care to CFH services. Faced with transition to a service where they will see a different CFH nurse at each consultation, women often transfer their care to a GP expecting to receive continuity of carer.

I just hear a lot of our women saying that they don’t want us to discharge them because they’re used to coming and seeing their known midwife and then they’re going out into the community to CFH nurses who they don’t necessarily know and they’re seeing different CFH nurses at every visit…so rather than going to CFH nurses women are going to their GP for that sort of care. [Midwife ACM]

CFH nurses reported using the initial visit with a family as an opportunity to engage and develop trust on which to build a longer-term relationship and to provide ongoing support to families.

You also create trust, and when you’re talking about some of the mental health issues… that’s part of the relationship. I’ve been doing this for 30 years and the more you see the client the more you can develop the skill to understand. Even if they say nothing, they walk in and you know there’s a problem. They have trust with you, and that builds, and that is what is really important. [CFH nurse MCAFHNA]

General practitioners also emphasised continuity of carer, referring to ‘the medical home’ ^a^. GPs described their relationships with families as ideally spanning time from the antenatal period, throughout pregnancy into the postnatal period, and thereafter. One GP participant defined continuity as seeing the family over repeated visits.

Overriding everything is the relationship you have with families and to make sure that they feel comfortable about coming into the surgery to ask for care especially the children who need to feel that the GP is part of their family. [GP]

Regardless of the philosophy of the general practice in which they worked, PN readily described the importance of developing relationships with families.

I feel that I play an incredibly important part, particularly in the early years. One, we’re ideally placed to get to know the parent and the child, particularly if you’re seeing that child regularly for immunisations. [PN APNA]

The professionals perceived that developing a relationship takes place over a period of time. However, this timeframe was bounded by their contact with the family within their service setting rather than the broader maternity and CFH service context. Professionals valued relational continuity as in seeing families over time they can assess how they are functioning. Familiarity increases the likelihood that the professional will provide the support required by individual families. Families develop trust in the professional and are more likely to access the service regularly. This in turn increases professionals’ capacity to provide appropriate parental support, developmental surveillance and health promotion activities to prevent adverse outcomes for children and families.

### Communication pathway; informational continuity

Communication pathways refer to the methods used by organisations and professionals to transfer client information, either via written communication (hard copy or electronic) or verbal (face-to-face or telephone conversation). All groups considered communication to be essential in achieving continuity. Ineffective communication procedures between organisations and professionals impacted greatly on the practice of each of the professional groups. Professionals often reported experiencing difficulty establishing relationships with families (*relational continuity*) due to a failure in communication. Communication pathways described by participants could be categorized into either *information transfer processes* or *person-to-person.*

*Information transfer processes* refer to procedures for transferring information from one service to another. Participants described various types of *information transfer*. ‘Traditional’ procedures included hard copy birth notifications, immunisation registers and personal health records.

…we have a state-wide service as well. The midwives put in their birth notifications through a central service and that is forwarded out through the different regions to the CFH nurses. [CFH nurse MCAFHNA]

Alternatively *‘band-aid’ procedures* or ad hoc strategies were developed to improve traditional procedures that had been found lacking. Several ad hoc systems had been developed to facilitate improved communication between professionals e.g. secure electronic messaging and additional hard copy documentation were reported as helpful in facilitating timely transfer of client information.

we have… priority information form that we ask the midwives …. to fill out to send to us as soon as they can, and just prior to us going out and doing our visits with the family. And that has been actually helpful and I think it’s helped the midwives recognise the information they have. [CFH nurse MCAFHNA]

P*erson-to-person* communication between two professionals may occur via face-to-face, via phone conversation or email communication. This procedure was implemented to support deficient *traditional* communication processes.

I have a number of private practitioners who just send me emails. We do it on a relatively de-identified basis. It’s just - because the person’s there that afternoon…I know that (a) the person’s turned up to the appointment, (b) that there’s been some sort of outcome, so that when I do see the person again, I can say, ‘oh look, how did you get on with the psychologist, whoever it is’. [GP RACGP]

Alternatively professionals instigated *person*-*to-person* communication to enable the development of a relationship with another professional.

*Effective* communication was important to each of the professional groups, i.e. when all relevant information was passed onto the service in a timely manner either through *traditional* or *band-aid* communication. A timely manner referred to information arriving in time for the professional to utilise it in supporting the family. Professionals believed that timely information transfer resulted in several positive outcomes both for themselves and for families. They indicated that families felt more supported when professionals were informed and were up-to-date with their history and current issues.

We’ve had a bit of a breakthrough in this respect … Maternity services adopted the program obstetriX… and through that we’ve just started receiving discharge summaries… and those discharge summaries include the antenatal psychosocial assessment information and also the antenatal Edinburgh scores… And now, when we see clients and childhood health, we’re able to say, ‘look this is the information that maternity services – your midwife – has passed onto us.’ [CFH nurse MCAFHNA]

Informational continuity was particularly important to the CFH nurses who practise under a continuity of service model and were not familiar with the family. This avoided the situation where families were required to retell their stories to several health professionals. This first excerpt demonstrates the CFH nurse’s frustration at being the person who requests a family repeat their story, while the second demonstrates the midwife’s understanding of the families’ perspective.

But I still think the midwives are a bit reluctant to forward information… the information you sometimes get in their blue books or in child health records, it might just have ‘caesarean’… yet there’s a whole other story behind that when you start talking to the mothers …midwives having a conversation and the mothers give them the information and then we turn around and turn up the next day and ask for that same information again. [CFH nurse MCAFHNA]

I think for the families the spin-off from all of that is that they feel like they’re retelling their story so many times and sometimes really important stories need to be passed on. [Midwife ACM]

Maintaining informational continuity influences the developing relationship between the service and family. Where there is continuity of carer, nurses have an opportunity in subsequent visits to develop professional credibility with families should the initial consultation be problematic, whereas nurses working within a continuity of service model may never see some families again.

Communication was described by professionals as ‘poor’ when information was either insufficient or late arriving, compromising the outcomes for families and therefore obstructing their practice. Poor communication resulted from a failure in traditional communication procedures. Outcomes were perceived as being compromised if professionals were unable to adequately support families’ needs. CFH nurses were left feeling unsure of families’ histories, arrangements made for them and further management required. CFH nurses felt responsible when problems with information transfer occurred, as they believed families saw them as the current face of the health system. The breakdown in information transfer was viewed as inefficient and careless practice.

We’ve had issues with the discharging hospitals who appear to have absolutely no knowledge of our service, which I find quite alarming. It’s the prem babies and the multiple births, and it’s really the lack of information from the midwifery sections in those hospitals, and understanding the imperative of that link on discharge. That’s something that needs to be addressed. [CFH nurse MCAFHNA]

As they believed this failure might reflect on their own professional identity, there was a significant level of criticism of colleagues in other disciplines:

The process would be better if the midwife-at-home service actually rang us and talked to us about the client, so that there was some continuity, so that they could actually speak to us instead of it being a piece of paper, which depends on someone typing the right things in the right places. [CFH nurse MCAFHNA]

Several CFH nurses recognised that it was the system failing rather than a failing of an individual professional. They indicated a willingness to work more closely to improve communication between services.

### Co-ordination of care; management continuity

Continuity for women and families also relies on management continuity which means the co-ordination of care. Participants perceived that management continuity centred on the *co-ordination of care* within their own service setting rather than the broader maternity and CFH service. Each professional group perceived themselves as the best positioned to co-ordinate care for the family. Midwives working within continuity of care models perceived themselves as the most appropriate professional to co-ordinate care from antenatal through to the postnatal period.

…just by having that one person who coordinates care and is able to hand over that story to child health nurse or to the GP makes an incredible difference …my past experience - knowing the difference that it makes compared to trying to pass on information in a fragmented system. [Midwife ACM]

Midwives argued that models of care that provide continuity of midwifery care to six weeks postnatally, can help to address the current gap experienced by women and families between discharge from maternity or midwifery service and the initiation of the CFH service.

they didn’t hear from the CFH nurses often for two weeks in terms of linking them in with the postnatal groups that they would have. So the women had already done the toughest patch of their postnatal time with their babies…The midwifery service didn’t spill over to provide that area and the child health nurse didn’t kick in soon enough. [Midwife ACM]

Similarly, CFH nurses saw themselves as the constant support for the family following birth, with the other services (e.g. allied health, general practice) being accessed as required. They considered that providing longer term support and the ability to link families to other health professionals according to their needs were crucial in providing continuity. Participants perceived that providing continuity enabled them to monitor each family’s physical and psychosocial wellbeing over time, resulting in better outcomes.

There are a lot of services around the edges or linked in but it is up to us to get them involved. The CFH nurse is the one that sees the mother or is the coordinator because we look at the whole picture. We’re looking at their mental health as well the feeding and development of the baby…the other services are just looking at one section. So we see them over a longer period of time, I think we do coordinate most things with the mother. Some of the others agencies may be in for the shorter term and then pull out because things are going well. [CFH nurse MCAFHNA]

GPs also believed that they were the most appropriate professional to co-ordinate care for children and families via the development of a long term relationship. GPs described themselves as having the potential to favourably affect family outcomes.

The doctor’s relationship may span the time, prior to pregnancy, during pregnancy and thereafter… has the potential to favourably affect the health of the child, the parents and family dynamics by reducing risk factors and promoting protective factors with a view to collaboratively building resilience. A real strength of the general practitioner is the ability to be responsive to individual needs rather than impose a one size fits all intervention. [GP RACGP]

Practice nurse participants supported general practice as the most appropriate service to co-ordinate care for families. They also saw themselves as playing an integral role in that co-ordination of family care; several had strong views on health promotion and prevention, especially around parenting.

…some of the basic things would be assessment of wellbeing of children in their growth and development and vaccinations, assessment and treatment during illness or following injury. We provide support, health care and education to the carers, both for physical care and also for behavioural support. In that, we’re also assessing the coping levels of parents and carers. So, if we need to refer them on for further services, then we can do that. Another thing … we do quite often is assess and advise over the phone to parents, regarding illness, emergencies and vaccines. [PN APNA]

Each profession group thus maintained that developing a relationship with the family was important for preserving continuity.

## Discussion

This paper aimed to examine the concept of continuity across the maternity and CFH service continuum from the perspectives of professional leaders from different disciplines. All professional groups valued continuity as the ‘ideal’ for all families and children. However, the term continuity was perceived differently by each of the professional groups, with most participants primarily referring to continuity as occurring within their own service rather than across or between services. Midwives perceived continuity was achieved through continuity of carer, as did GPs. For CFH nurses, the organisational emphasis of ‘continuity of service’ differed from some participants’ personal philosophy of care or their personal preference to provide continuity of carer.

Management continuity bridges gaps in the healthcare system, facilitating care which the client perceives as connected and coherent [48]. Our study found little evidence of co-ordination of care across CFH service providers. Each professional group was primarily concerned with the management of care within the confines of their own service (maternity service, CFH nursing service, general practice) as opposed to management continuity between services or professional groups. Notably practice nurses spoke from the perspective of their employers, general practitioners, rather than from a PN perspective.

The main referral process between professional disciplines occurred from midwives either to CFH nurses or to GPs. Referral between CFH nurses and general practice rarely occurred. Each group readily referred families to secondary services (social work, speech pathology, psychology, paediatrician). In terms of managerial continuity, each group saw themselves as the most appropriate and best qualified to coordinate a family’s care. Therefore, management continuity was confined, in most instances, to referral to secondary services. This finding was particularly highlighted by CFH nurses and general practitioners since these services spanned a longer period (from birth to 5 years or in the case of GPs from pre-birth to adulthood). Consequently the development of interprofessional relationships between maternity and CFH nurses, GPs or PNs rarely occurred.

This limited opportunity to develop interprofessional relationships resulted in a lack of understanding of the contribution and role other professional groups played in delivering services to women and families. Unfamiliarity, together with poor communication and information transfer between health professionals and services, resulted in professional territoriality [49]. This made collaboration between services problematic. In the past CFH nurses visited maternity units to introduce the service to new mothers [50]. However, this has changed; CFH nurses no longer have direct access to new families but rather depend on referral from other health professionals [51]. Families rarely connect with CFH nursing services without receiving information about the service. This is not the case for midwives and general practice, where families actively seek these specific services [52]. Families actively connect with them either for pregnancy care (midwifery services) or in relation to episodes of illness (general practice). However, GPs reported difficulties with referral processes between themselves and other professionals as a major barrier to continuity. They complained that they rarely received feedback after they referred families to other professionals.

Discontinuity was most evident in the lack of informational continuity between services. Professionals described processes which were inadequate for linking services. A range of ad hoc strategies were developed to improve informational continuity due to ineffective and often incompatible communication systems. Informational discontinuity leaves professionals uninformed and ill-prepared to support clients. For women and families, it can result in not being linked to services and being left unsupported in the early postnatal period.

Participants identified effective communication was seen as crucial to developing a relationship with families, which facilitated a smooth transition of care across services (continuity). However, in some instances, informational continuity also facilitated the development of relational continuity. The level of importance of each dimension depended on the situation. For example, in the first home visit CFH nurses place most emphasis on having access to past health history (informational continuity) and developing a relationship with the family (relational continuity), whereas in subsequent visits they also distinguished developing a care plan with the family (managerial continuity).

In this study professionals emphasised the first contact visit with families as crucial to facilitating initial engagement for the purpose of developing a relationship. Other researchers have reported the value of the first contact in this regard [16]. CFH nurses working in a ‘continuity of service’ model, perceived the first visit as particularly important as they might not have subsequent opportunities to enhance the relationship subsequently and therefore needed to engage the family in the service at first contact. Whereas if a family experienced problems linking with the service but they were able to continue seeing the same CFH nurse (continuity of carer model), it was purported that additional opportunities to develop a relationship with the family would eventuate. Each professional group viewed the development of a relationship with families as paramount. They all believed that a trusting relationship aided them in their efforts to co-ordinate care for the family and facilitated continuity of care.

The advantages of continuity for health professionals have been reported. Appleton and Cowley [53] reported CFH nurses’ preference to work in a ‘continuity of carer’ model as it facilitated their ability to undertake accurate assessment of a family’s health needs. GPs have reported that continuity facilitates the development of essential skills over time, such as the ability to make an individual multidimensional diagnosis [54]. However, providing access to ‘continuity of carer’ is becoming increasingly difficult given the shortage of doctors in some areas, closure of practice books and reorganisation into larger group practices [35,55,56]. The RACGP *Curriculum for Australian General Practice*[57] promotes integration across disciplines, developing partnerships with consumers and community organisations, and evaluation as key to the population health approach.

The importance of working alongside parents, recognising their expertise through a strength-based approach to practice has been incorporated into the CFH nurse curriculum [58]. Policies have encouraged CFH nurses to participate in Family Partnership Model training [59,60] to develop knowledge and skills to enable them to effectively support parents. However, as services have moved from an individual family-focused philosophy of care to a population health service approach, CFH nurses have developed skills and implemented strategies geared towards influencing determinants of health. This has placed additional demands on CFH nurses over recent times [61]. Early postnatal discharge within two days of birth in most areas has increased this burden, as new mothers require support to establish breastfeeding and develop parenting skills at home [62]. There is also increased pressure on CFH nurses to provide a universal home visit to every family within the first two weeks following birth [16]. In addition, the social and cultural diversity of the client population has changed, challenging the CFH nurses’ skills in supporting families from a range of backgrounds [63]. While the system supports the education of CFH nurses to work in partnership with families, increased workload and resource shortages create system barriers that impede the CFH nurses implementing this approach [16,63]. Many CFH nurses find it difficult to balance an individual family-focused philosophy of care with a population health approach, resulting in their having to prioritise families by risk [16]. This often leaves families without identified risk without the support they may require. Fragmentation and discontinuity adversely affect the quality of care provided to families, risking dissatisfaction and disengagement from the service.

Full integration of services across the public-private divide may not be achievable or necessary to provide a CFH continuum of care. The development of a range of models of practice to support families may well be the answer. Irrespective of what a model of practice looks like, deliberate decisions on how best to provide care to all families is required. The roles and responsibilities of each professional in service provision require clarification to ensure a smooth transition across services. Further, a commitment to adequate resources to facilitate collaborative working is essential to support the efforts of professional groups. A move towards developing collaborative practices will require committed action from each professional group, and a significant change in the professionals’ vision of the service.

### Limitations

Our purpose in sampling representative leaders of each professional group was to gain the perspective of that group on current service provision across the maternity and CFH service continuum. However, as participants either self-selected or were nominated by professional peers, we cannot be certain that the perspectives provided by these individuals were representative of their professional groups. We also experienced some difficulty recruiting GPs into the study. GP participants from the general practice special interest group did participate and numbers were limited compared to the midwife and CFH nurse professional groups. Further, PN predominantly provided perspectives reflective of their employing GP rather than representing their own professional group.

However, the perspectives offered by all professional groups around the identified themes were congruent. Despite these limitations this study is the first to canvass the views of all universal/primary care providers and it provides increased understanding of continuity within the maternity and child and family health services continuum of care.

## Conclusion

The aim of this paper was to examine the concept of continuity from the perspectives of midwifery, CFH nursing, general practitioner and practice nurse (PN) professional leaders. Although the term ‘continuity’ was perceived differently, perspectives on the importance of continuity for all families and children were similar in each of the professional groups, despite a variance in funding arrangements, resource difficulties and work environments. However, while each group professed to be working on behalf of families, the focus of their practice appeared to be on sustaining what they traditionally perceived as the boundaries and primacy of their service. Service models remain organised around the requirements of health professionals rather than the needs of women and families.

The problems identified as contributing to the fragmentation of the CFH continuum of care are difficult to unravel. The overlapping concepts discussed in this paper provide some insight as to how professional groups might begin to address these problems. However, additional effort to work more collaboratively, particularly around the point of transition of care between services is vital if service gaps are to be addressed.

## Endnote

^a^ A medical home is defined as a regular doctor or source of primary health care that is easy to contact by phone during office hours, always or often knows the person’s medical history, and always or often helps coordinate care from other doctors or sources of care.

## Competing interests

The authors declare that they have no competing interests.

## Authors’ contributions

KP: participated in the research design, data collection, completed the drafting of the paper, VS: conceived and participated in the study design and paper focus, help draft the paper and provide critical review and guidance. CF: participated in the study design and paper focus, help draft the paper and provide critical review and guidance. SK: participated in the study design and paper focus, help draft the paper and provide critical review and guidance. All authors read and approved the final manuscript.

## Pre-publication history

The pre-publication history for this paper can be accessed here:

http://www.biomedcentral.com/1472-6963/14/4/prepub
